# The Health of the Royal Navy

**Published:** 1862-07

**Authors:** 


### Art. VI.—THE HEALTH OF THE ROYAL NAVY.

"The first and most important question to the seaman is his health," said Lord Clarence Paget, in the debate on the Navy Estimates during the present Session of Parliament, and he added :—" The death-rates for the fleet are in general satisfactory ; but in some exceptional cases they are not, ranging, in fact, as high as 60* per 1000 per annum. On home stations the rate is 10, and the average on the whole is 16. We have inquired whether some improvement might not be made in the food of the seamen, and also in the ventilation of the berths. We have had a Committee sitting some time, and the result is that the Admiralty have resolved to make every possible effort to improve the ventilation of the ships. Everybody who has been on board ship in the lower deck, will know that the atmosphere is sufficiently bad to provoke almost any kind of disease, especially phthisis and fevers, as has been shown by the returns from the Mediterranean fleet. We propose, therefore, in the large ships of the line, to reduce the complement of men from 880 to 800; in large frigates, from 570 to 510; and in the small frigates from 350 to 310. I advert to this merely to say that the principal reason for reducing the complement of seamen is that the ventilation may be improved. With regard to food, the House last year agreed to allow an establishment at Deptford for the curing of salt beef. This has been of the greatest comfort and advantage to the seamen. It has cost money, no doubt; but depend upon it, it is money well spent, as the men and officers now get good corned beef, instead of the 'old mahogany' of our younger days."

It is so rare for a Government department to take the initiative or to exhibit any active interest in the hygienic condition of the individuals under its control, except under the pressure of public opinion or the sharper incitement of some great catastrophe, that, in welcoming an exceptional fact of this nature, it may seem a little ungenerous to cavil at the terms in which it is announced. And yet, whatever pleasure we may experience in the knowledge that the great question of the health of the seamen of the Royal Navy is fully present before the attention of the Admiralty ; whatever gratitude we may feel for the important measures already taken or under consideration for bettering the sanitary condition of our ships ; and however much we may be disposed to attach the fullest meaning of which the words are susceptible to the statements of Lord Clarence Paget : when we compare these statements with, or test them by, the great modifications which have been going on in the construction of the Navy within the past two years, and are still in progress, it is impossible to avoid an unpleasant suspicion that the Admiralty has not yet fairly appreciated the real significance of the question at issue. The subject is one which, although at all times of the greatest moment to the public welfare, has now assumed unusual interest and importance in consequence of the changes unhappily forced upon our naval administrators by the events of the civil war in America.

It is not a little curious that notwithstanding the many discussions in Parliament, and, it may be added, notwithstanding all that has been said or written in the public press, of the capabilities of armour-clad ships as war vessels, their character as dwellings, and probable influence upon the health of their crews, does not appear to have ever been systematically discussed. Now, the defective sanitary condition of our wooden ships of war, more particularly as to the ventilation of the lower decks, would, it might be conceived, have suggested the propriety, nay, the necessity, at the very outset of so greata modification in the Navy as the construction of armour-clad vessels, of considering their health as well as their fighting qualifications. But when it is considered that hitherto armour-clad ships have been so built that their walls will, it may reasonably be inferred, rapidly accumulate and store up heat; and that the circulation of air in the between-decks, unless artificially provided for, will be diminished to the least amount, in consequence of the smaller number and less size of the port-holes, and the rigid exclusion, for the sake of strengthening the walls, of all apertures not absolutely required between the exterior and interior of the vessels, it is manifest that, unless the evils likely to arise from these sources are specially provided against, these ships may in the end become veritable pest-houses. That this foreboding is no mere sanitarian exaggeration, and that we are justified in fearing, there being no evidence to the contrary, that the health condition of armour-clad ships has not received that attention which the importance of the subject demanded and still demands, may be gathered from the results of the trial trips of the vessels of this class already built. They have proved to be very uneasy in a sea-way, and as a consequence it may be surmised that it has been necessary to keep the ports closed to a greater extent than in wooden ships of war—and closure of the ports, it is not to be forgotten, cuts off almost the entire ventilation of the between-decks. The heat of the between-decks, so far as may be judged from the experiments recorded in the naval news of the day, would seem to range higher than in wooden steam-vessels of war—the sickliest ships in the service, in consequence of their heightened temperature. The sailors complain of the extreme "wetness" of the armour-clad ships; and it is highly significant as to the state of the between-decks, that a little while ago a report was current in naval circles that deck houses would probably be needed for the crews. Finally, experience has taught that the bilges of these ships require to be dealt with after a new fashion. We learn from the *Times* of the 10th ult., that some seventy tons of bricks and mortar have been built into the bottom of the *Warrior* to prevent the lodgment and consequent stagnation of the bilge-water. The numerous valves which are inserted in the lower portions of the various sections of the *Warrior* are, it would appear, necessarily shorter downward than the divisions; or, in other words, the valves cannot conveniently be made to descend to the angles in the bottom of the ship. Now, the bilge and waste water which becomes stagnant is that which lodges in the bottom between the sections, where it remains undisturbed, and soon becomes putrid. Here it is that the masonry has been applied. All the leakage and waste water will by this alteration be brought to one common level, and also to one common suction. Additional pumps and valves have also been added; and by these arrangements it is expected that scarcely a gallon of water can hereafter accumulate in any part of the vessel. It is to be hoped that the absorbing power of brick and mortar has not been overlooked and provided against.

But armour-clad ships are one-decked vessels; hence the problem of their ventilation and sanitary condition is doubtless simpler and ought to be more readily met than that of several-decked ships. The fighting and sailing qualities of ships of war must of necessity be always primary considerations with naval architects, but this does not in any degree imply the need of a neglect of the sanitary condition. As with house-architects the health as well as the living condition of a house ought to be an imperative consideration, so with naval architects, the health condition as well as the fighting and sailing qualities of ships should be also imperative. The former condition is just as sound a matter for scientific discussion and argument, and as important ultimately to the public and the service, as the latter conditions. The absence of any indications of the question being dealt with systematically and scientifically during the discussions and preliminary steps pending the present reconstruction of the navy, and the facts mentioned, have naturally given rise to the doubts we have expressed. In the construction of a ship of war it cannot be questioned that its health condition ought to enter as largely into the scientific consideration of naval architects as its fighting and sailing qualities. These latter conditions are of primary importance only in so far as they are the original objects of and must determine the form of the vessel, and modify (not set aside) the circumstances for the solution of the health problem. House architects know that it is easier to build a new house with due regard to its sanitary condition than it is to remedy sanitary defects in an old one. This rule we fancy ought to hold good with ships. The Admiralty is entirely responsible to the public in this matter. It lacks nothing, either in men or means, for dealing with the health condition as well as the fighting and sailing qualities of ships in the most effective manner. We hail with pleasure the signs of interest it has already manifested in the subject, as evinced by their spokesman Lord C. Paget, but that interest is wanting in thoroughness. It has not shown itself a moment too early, but rather too late. Letting bygones, however, be bygones (to use the time-honoured phrase), we look to the future. The Horse-Guards was taught its lesson of thorough sanitary reform by the horrible disasters in the Crimea in the winter of 1854-55. It will be well for the Admiralty to have a care lest it be taught its lesson of thoroughness by some equally horrible catastrophe. What would be the lot of the crews of a fleet of our Ironsides in the yellow-fever zone (no unlikely destination with the present aspect of affairs in the West), if that dreaded plague got among them, and the health-condition of the ships was such as we have reason to surmise it may be? The probable results are dreadful to contemplate. Here is a gauge from the preface to the Reports of the Health of the Navy in 1858 (published October, 1861). Yellow fever was introduced (according to Dr. Bryson) into the *Icarus*, one of the West India squadron, in September, 1861. She subsequently infected the *Imaum*, the *Hydra*, and the *Barracouta*. The crews of these ships numbered about 640 men. Of these, in the course of six weeks, no less than 160 were seized with yellow fever, and *seventy* died. It may be noted also that fully one-half of all the deaths from fever throughout the service in the three years 1856-58 arose from yellow fever.

The following observations on this pestilence, by Dr. Milroy, in a recent letter to the Right Hon. Sir John Pakington,* are not without significance in connexion with the foregoing remarks:—

"This very fatal form of fever," he writes, "appears to have been at times more destructive in our ships of war within the last fifteen or twenty years than was ever known before. *All the vessels which have been smitten most severely have been steamers.* No sailing ships in former times seem to have sustained such terrible losses as the *Eclair* in 1845, the *Dauntless* in 1852, the *Malacca* in 1856, and the

* *The Health of the Royal Navy considered*, in a Letter addressed to the Right Hon. Sir John S. Pakington, Bart., S.C.B., M.P. By Gavin Milroy, M.D., F.R.L.C., Medical Commissioner to Jamaica in 1851, and Member of the Sanitary Commission to the Army in the East in 1855-56. London: 1862.

*Icarus* in 1860. This fact is very suggestive. That the excessive heat on board, without adequately free ventilation, has had much to do with the increased malignancy of the distemper is more than probable. It would seem, too, that the accumulation of dirt and other offensive rubbish under the machinery, etc., has in several instances added not a little to the impurity of the close hot-air in the between-decks. Reference is frequently made in the surgeon's reports to both these evils." (p. 32.)

In one sense it is no doubt true, as Lord C. Paget says, that "the first and most important question to the seaman is his health;" but the question is even of more importance to the public and the Admiralty than the seaman. If we had to depend upon the seaman himself, proverbially reckless, for such care as was requisite for the preservation of his health, it is to be feared the crews of our ships and efficiency of the service would fare badly. But he has no control over either the construction or health-condition of his dwelling, his food, his clothing, or his exercise. In all these respects the Admiralty is the sole arbiter, and with it of course rests almost the entire responsibility of the sanitary condition of our ships and their crews. Hence it would have been better had Lord C. Paget's expression run—"the first and most important question to the Admiralty is the seaman's health." This both the public and Parliament seem too apt to forget or lose sight of except when the subject is forced upon them by some awkward or unfortunate event. If it were made incumbent upon the Admiralty in preparing the Naval Estimates to show the probable pecuniary loss to the public from failure of service by reason of sickness in the Fleet, perhaps the dry statistical details of the Health of the Navy would attract more attention, and point out a legitimate method of economy not often considered—a method which, while curtailing expenses, would actually conduce to greater efficiency of this branch of the public service. The sailor is a costly necessity, procured and perfected with too great difficulty to be lavishly expended.

Of the influence of sickness upon the efficiency of the Navy, Dr. Milroy has admirably observed, in the letter to which we have already referred, (and which we would commend to all our readers who are interested in this important subject, and who are wishful to learn the pith of the matter from one singularly well-qualified to write with authority upon it) :—

"It is now admitted, on all hands, that the strength and virtual efficiency of an armed force, whether afloat or on shore, is to be measured, not by the mere number of the names on the ship or regimental roll, however complete may be all the material equipments of the force, but really and truly by the actual number of hearty, vigorous men who are, from day to day, and from month to month, continuously available for fatigue duties of all sorts. Every man put on the doctors' list is so much power withdrawn from the full effectiveness of the living machine. Nay, it is more than this; for each such loss becomes the occasion of extra duty being cast upon the workmen to supply the void; and then, too, there is the time and labour of those who have to act as the attendants upon the sick to be taken into account. These consequences become a serious matter when sickness prevails to any considerable extent among a ship's crew. The energies of the workmen are over-taxed, their hours of mealtime and sleep are interfered with; continued extra fatigue creates weariness and discontent; and this is the very state of system in which health is liable to suffer from influences which it has hitherto resisted. Sickness thus gives rise to sickness in more ways than one; and this, too, is apt to go on in a progressively increasing ratio. Obviously, therefore, the necessity of averting or preventing to the utmost all disease, and of preserving, as far as can be done, uninterrupted health among a ship's crew, cannot be over-estimated as one of the main objects to be aimed at by all who have at heart the duty of maintaining a powerful navy, ready at all times for the defence and honour of our country. And the importance of the subject is yearly becoming greater, from the acknowledged difficulty that has often been experienced in recent times, of promptly manning a large fleet upon an emergency in consequence of the enormous demands of our mercantile marine for able-bodied hands in these days of increasing commercial activity." (p. 1.)

The average daily rate of sickness in the Navy for the three years 1856—58, varied from 46 per 1000 men on the Australian Station to 93 on the East India and China.* Throughout the entire force the sickness-rate averaged a little above 60 per 1000 in this period. In 1856, with an aggregate mean force of 51,730 men, the average number continually off duty from sickness was 3132—that is to say, a seventeenth part. In 1857 the daily sick-rate was about the same as in the previous year, in a force of 42,470; and in 1858, in a force of 43,120, the daily sick numbered 2859. In the three years the total number of days' sickness

| * STATIONS. | Mean strength for the three years. | Average daily Sick-rate for the three years. |
|---|---|---|
| East Indian and China | 7263 | 93 per 1000 men. |
| Irregular | 7117 | 69 ,, |
| West Coast of Africa | 1707 | 68 ,, |
| Cape of Good Hope | 1030 | 59 ,, |
| North America and West Indies | 4365 | 58 ,, |
| East Coast of South America | 1323 | 55 ,, |
| Pacific | 2387 | 53 ,, |
| Mediterranean | 7867 | 52 ,, |
| Home | 11,814 | 50 ,, |
| Australian | 437 | 46 ,, |

would be equivalent to about 23 days off duty annually by every man in the force.

The daily sick-rate on the Home Station in the same period averaged 50 per 1000 men ; and the total number of days' sickness was equal to 19 days' unfitness for duty annually for every individual in the force. This amount of sickness contrasts favourably with that experienced among the provident classes pursuing outdoor occupations, requiring great exercise, according to Mr. Neison's calculations. These show a rate of sickness per annum, within the Vicennial period, 21—40, of somewhat more than 3 weeks (3·7). On the other hand, the daily sick in the metropolitan police averaged but 30 per 1000 in the five years 1852—56, and in the city police 20 ; while the daily average in the Army stationed at home was, in 1859, nearly 14 (13·9), the mortality in that year being—it is well to remember—reduced to one-half of the rate which had existed among the troops three years previously.

The mortality from *disease* in the Navy, in the three years 1856-58, ranged from 3·6 annually per 1000 of the force on the Pacific station, to 37·7 on the East India and China station. Two of the three years in the latter station were years of war. The general average for the three years of deaths from disease, was a fraction above 15 per 1000 ; of deaths from all causes, 20. Rather less than one-fourth of the mortality arose from injuries, drowning, and suicide.

The annual death-rate from *disease* on the Home Station, for the period referred to, was 7·8 per 1000 ; from all causes, 10·3. The rates of mortality per 1000 per annum, of the male civil population at Navy ages, is, in town and country, 9·2 ; in the country alone, 7·7 ; and in one of the unhealthiest towns, Manchester, 12·4. The death-rates of the Navy on the Home station, thus contrast favourably with those of a civil population, even in the healthiest districts. In all comparisons of the Navy and Army death-rates with others, it is, however, not to be forgotten that the former are diminished in amount by the constant removal of weak and sickly men from the service by invaliding.

The average annual rate of *invaliding* in the three years referred to, was 31·5 per 1000 of the force ; and the permanent loss to the Navy from death and invaliding, was 51·7. In round numbers 1799 men were lost to the service by death and invaliding in 1856, out of an aggregate strength of 51,730 ; 2404 in 1857, out of a force numbering 42,470 ; and 2878, a loss equal to the combined crews of three of the largest line-of-battle ships, in 1858, out of a force of 43,120.

The invaliding returns it must be confessed neutralize, in no small degree, the pleasant impression which might be created by the seemingly low rate of mortality from disease. The loss to the

Navy from invaliding is as absolute as the loss from death. Let us, however, keep to the death-rates of the Fleet as they stand, and inquire if, as Lord C. Paget stated, they are "in general satisfactory." It is true that since the close of the great Continental war, the mortality in the Navy has undergone a marvellous reduction. Sir Gilbert Blane estimated the annual death-rate in the last three years of the war at 33 per 1000—a rate, by the way, but half that which had prevailed in the Fleet fifty years previously. But if we compare the death-rates of 1856-58 with those of 1837-43,* we shall find that in this respect matters appear to have been well nigh stationary within the period. The general average of 1837-43 is less than that of 1856-58; but the difference is not, perhaps, greater than what may be explained by fighting in India and China. In the former period the mortality was higher in the Mediterranean, and astoundingly so on the West-African Coast, as compared with the latter period; but on the Home, the South American, and the East Indian stations the death-rates were actually less. Doubtless these differences are susceptible of an explanation in favour of 1856-58; but no explanation will probably do away with the fact, that there has been no very marked improvement in the health condition of our Navy, so far as this may be gauged by mortality, since 1843; or else what improvements have been carried out have been neutralized by the greater sickness of the steam fleet constructed since then. Again, although the death-rate of the Navy on the Home station from disease, is less than that of the healthiest districts of the kingdom, setting aside the fact of invaliding, we are not too hastily to conclude that, therefore, this death-rate is satisfactory. The sailor has advantages for the maintenance of his health, which

| * STATIONS. | Mean Death-rate per 1000 strength annually from *disease alone* in the seven years 1837-43. | Mean Death-rate per 1000 strength annually for the three years, 1856, 57, 58. | |
|---|---|---|---|
| | | From Disease alone. | From all causes. |
| Home | 6·8 | 7·8 | 10·3 |
| Mediterranean | 10·7 | 8·4 | 11·6 |
| North America and West Indies | 19·2 | 19·8 | 24· |
| Brazilian | 6·7 | 21· | 25·2 |
| Pacific | | 6·9 | 8·7 |
| West African | 57·0 | 14·3 | 20·5 |
| Cape of Good Hope | 11·0 | 10·2 | 16·3 |
| East Indian and China | 34·2 | 37·7 | 47·6 |
| Australia | | 3·6 | 9·9 |
| Irregular | | 8·3 | 12·4 |
| Total | 14·9 | 15·2 | 20·0 |

a civil population rarely possesses. Hence the health-condition of the latter is no fitting criterion of what that of the former should be. It may be gratifying to find that a death-rate previously higher than this criterion has been reduced to it; but this reduction was effected some years ago, and cannot rightly be laid to the credit of the present representatives of the Admiralty. We look for a much higher condition of health among sailors than among a promiscuous civil population, suffering from all the vicissitudes of trade, and exposed helplessly, as a rule, to sanitary evils which a sailor ought never to be subjected to. Moreover, it is admitted that the present health-condition of our seamen is maintained under circumstances most detrimental to health ; and the returns before us show that these circumstances may be looked upon as the chief fostering causes of the diseases which most affect the efficiency of the crews.

The three great sources of sickness and death on shipboard are (1) Fevers ; (2) Diseases of the Respiratory Organs ; and (3) of the Digestive Organs.* Lord Clarence Paget has already told us in what manner these diseases are promoted. It is the same story on shipboard as in barracks and over-crowded dwellings. But does it not seem like a *charivari* of hygienics when at one and the same time we are gravely told that death-rates are "generally satisfactory," and yet that active measures are about to be taken for the removal of conditions upon which those death-rates to a great extent avowedly depend ?

Let us hope that the sanitary measures initiated by the Admiralty will be carried out as effectively, and with as much success, as those which have been introduced into barracks and military hospitals by the Commission on the Sanitary Condition of the Army.

*Apropos* of the food of the seamen to which Lord C. Paget refers, it is important to note that scorbutic disease broke out in two of our ships on the North American station, the *Jaseur* and *Jasper*, in 1858. In the former ship, "for nine months the crew had been victualled only twenty-six days on fresh provisions ;" in the latter ship, while cruising on the coast of Cuba, the "crew were on two separate occasions nearly three months without any fresh provisions." These are surely very curious facts.

We postpone any consideration of the ventilation of ships until

---

* NUMBER OF CASES.

| | Fevers. | Disease of Lungs. | Disease of Digestive Organs. | All causes. |
|---|---|---|---|---|
| 1857 | 6,835 | 9,524 | 8,667 | 66,546 |
| 1858 | 14,779 | 10,678 | 10,467 | 75,924 |
| Totals | 21,614 | 20,202 | 19,134 | 142,470 |

the publication of the report of the Parliamentary Committee on the subject.

We cannot, however, resist the temptation, in concluding this article, to quote one illustration of what may be done for the welfare of our seamen and the benefit of the public service, by sanitary measures rightly conceived and carried out, even under apparently the most disheartening circumstances. The illustration leads us to hope that the excessive sickness and mortality, or rather the insanitary conditions on which these depend, of the East Indian and China and Brazilian stations will ultimately be found capable of being dealt with in an equally effectual manner. It will have been noted that the mortality from disease alone, on the West Coast of Africa, in the six years 1837-43, was no less than 57·0 per 1000 per annum. In 1856-58 the mortality was only 14·3. In what manner this remarkable diminution in the death-rate was brought about may be gathered from the following observations in the Health Report of the Navy for 1856 :—

"With a mean force of above 1680 men, there were only seven deaths from fever, being in the ratio of about a little more than four to the thousand, a mortality so small, compared with that of former years, that it was almost incredible, and might well lead to the belief that the coast, like some of the cleared portion of the North American Continent, is becoming healthy ; but with the exception of the non-appearance of yellow-fever, which does not depend on terrestrial emanations, the climate has undergone no salutary change. The seemingly interminable forests which cover the land in every direction, and the deadly swamps which fringe the estuaries of every tidal river, are still as prolific of the fever poison as they were in times gone by, when the death-rate in the squadron was ten times greater. How, then, it may be asked, are we to account for this improvement ? Simply by the change which has taken place in the mode of conducting the duties of the station. By a wise and humane regulation the deadly practice of sending boats away on detached service, to watch or intercept slavers, has been interdicted, or, at all events, greatly restricted. Prize crews are no longer turned adrift to wander through the streets of Sierra Leone, when the vessels they navigate from distant parts of the station are delivered up to the authorities of the Mixed Commission Court; the orgies of the 'barn,' which lowered the character of the white man in the eyes of the black, have long since ceased ; and last, though not least, the introduction of quinine wine as a preventive of fever has not only reduced the number of febrile attacks, but has lessened the severity of those which do occur, and thus the mortality has also been reduced to a level which does not materially exceed the death-rate from fever of the more healthy stations.

"There has also been a great change in the medical treatment of febrile disease. The so-called active measures which were in vogue but a few years since, have given place to others of a more rational character. Blood-letting is no longer carried to an extent which leaves the patient little chance of recovery when the fever terminates, and the rash and empirical use of calomel, in large and frequently repeated doses, to produce ptyalism, has been abandoned—not only on account of the impossibility of producing ptyalism while the fever lasts, but because mercury given to excess in any form has a most injurious effect on the constitution. If these changes have had no effect in reducing the mortality, they at all events have lessened the sufferings and misery entailed on patients who, though they survived the fever, lingered long in a state of debility from the effects of blood-letting and mercury." (p. 115.)

Many valuable illustrations of the value of quinine wine as a preventive of fever on the African coast will be found in the three Health Reports for 1856-57 and 1858.

---

